# Mutation of the human mitochondrial phenylalanine-tRNA synthetase causes infantile-onset epilepsy and cytochrome *c* oxidase deficiency^☆^

**DOI:** 10.1016/j.bbadis.2013.10.008

**Published:** 2014-01

**Authors:** Abdulraheem Almalki, Charlotte L. Alston, Alasdair Parker, Ingrid Simonic, Sarju G. Mehta, Langping He, Mojgan Reza, Jorge M.A. Oliveira, Robert N. Lightowlers, Robert McFarland, Robert W. Taylor, Zofia M.A. Chrzanowska-Lightowlers

**Affiliations:** a Wellcome Trust Centre for Mitochondrial Research, Institute for Ageing and Health, Newcastle University, Newcastle upon Tyne NE2 4HH, UK; b Child Development Centre, Addenbrooke's Hospital, Cambridge, UK; c Medical Genetics Laboratories, Cambridge University Hospitals NHS Foundation Trust, Cambridge, UK; d Department of Medical Genetics, Addenbrookes Hospital, Cambridge, UK; e Biobank, Institute for Genetic Medicine, Newcastle University, International Centre for Life, Central Parkway, Newcastle upon Tyne NE1 3BZ, UK; f The Wellcome Trust Centre for Mitochondrial Research, Institute for Cell and Molecular Biosciences, Newcastle University, Newcastle upon Tyne NE2 4HH, UK

**Keywords:** OXPHOS, oxidative phosphorylation, aaRS, aminoacyl-tRNA synthetase, mt-, mitochondrial, mtDNA, mitochondrial DNA, MRI, magnetic resonance imaging, LBSL, leukoencephalopathy with brain stem and spinal cord involvement and lactate elevation, PCH6, pontocerebellar hypoplasia type 6, MLASA, myopathy, lactic acidosis and sideroblastic anaemia, Mitochondria, Mitochondrial disease, Aminoacyl-tRNA synthetase, Aminoacylation, Mitochondrial translation, Protein synthesis

## Abstract

Mitochondrial aminoacyl-tRNA synthetases (aaRSs) are essential enzymes in protein synthesis since they charge tRNAs with their cognate amino acids. Mutations in the genes encoding mitochondrial aaRSs have been associated with a wide spectrum of human mitochondrial diseases. Here we report the identification of pathogenic mutations (a partial genomic deletion and a highly conserved p. Asp325Tyr missense variant) in *FARS2*, the gene encoding mitochondrial phenylalanyl-tRNA synthetase, in a patient with early-onset epilepsy and isolated complex IV deficiency in muscle. The biochemical defect was expressed in myoblasts but not in fibroblasts and associated with decreased steady state levels of COXI and COXII protein and reduced steady state levels of the mt-tRNA^Phe^ transcript. Functional analysis of the recombinant mutant p. Asp325Tyr FARS2 protein showed an inability to bind ATP and consequently undetectable aminoacylation activity using either bacterial tRNA or human mt-tRNA^Phe^ as substrates. Lentiviral transduction of cells with wildtype FARS2 restored complex IV protein levels, confirming that the p.Asp325Tyr mutation is pathogenic, causing respiratory chain deficiency and neurological deficits on account of defective aminoacylation of mt-tRNA^Phe^.

## Highlights

• The tissue specific variation in presentation of aaRS mutations is discussed.
• A further clinical presentation of a FARS2 defect.
• Molecular mechanism of FARS2 defect is characterised in vitro.

## 1 Introduction

Human mitochondria possess their own translation machinery in order to produce 13 mitochondrially-encoded polypeptides that are subunits of the oxidative phosphorylation (OXPHOS) complexes. The translation machinery has a dual origin and comprises mitochondrially (mt-) encoded transfer RNAs (mt-tRNAs) and ribosomal RNAs (mt-rRNAs) as well as numerous, nuclear-encoded proteins including mitochondrial ribosomal proteins, initiation, elongation and termination factors, a methionyl-tRNA transformylase and mitochondrial aminoacyl-tRNA synthetases. Hence, mutations in either the mitochondrial genome (mtDNA) [1,2] or the nuclear DNA [3] can cause defects in mitochondrial protein synthesis resulting in a variety of mitochondrial disease phenotypes affecting both children and adults.

Aminoacyl-tRNA synthetases (aaRSs) play a key role in the faithful translation of the genetic code since they catalyse the attachment of each amino acid to its cognate tRNA. The human proteome includes two sets of aaRSs that are encoded by nuclear genes and are involved in either cytosolic or mitochondrial protein synthesis [4], with the exception of *GARS* and *KARS* that function in both domains. Based on the tRNA recognition mode and the domain organisation, aaRSs are divided into two classes: Class I aaRSs (mainly active as monomers) and Class II enzymes (mainly active as dimers or tetramers) [5]. In recent years, recessively-inherited mutations in a growing number of mitochondrial aminoacyl-tRNA synthetases of both classes have been associated with a diverse spectrum of early-onset mitochondrial clinical presentations [6]. These include mutations in the genes for *DARS2* causing leukoencephalopathy with brain stem and spinal cord involvement and lactate elevation (LBSL) [7,8], *RARS2* causing pontocerebellar hypoplasia type 6 (PCH6) [9], *YARS2* causing myopathy, lactic acidosis and sideroblastic anaemia (MLASA) syndrome [10,11], *SARS2* causing hyperuricemia, pulmonary hypertension, renal failure in infancy and alkalosis (HUPRA) syndrome [12], *HARS2* associated with ovarian dysgenesis and sensorineural hearing loss [13], *AARS2* causing infantile cardiomyopathy [14], *EARS2* associated with leukoencephalopathy [15], *MARS2* causing neurodegenerative phenotype in flies and autosomal recessive spastic ataxia frequently associated with leucoencephalopathy (ARSAL) in humans [16], *LARS2* associated with premature ovarian failure and hearing loss in Perrault syndrome [17] and *KARS*, which encodes both the cytosolic and mitochondrial lysl-tRNA synthetases, and is associated with non-syndromic hearing impairment [18]. Recently, mutations have been reported in the *FARS2* (mitochondrial phenylalanyl-tRNA synthetase) gene in two families with infantile mitochondrial encephalopathy reminiscent of Alpers' syndrome [19,20]. In the present study, we report novel *FARS2* mutations including a large scale genomic deletion, in a child with muscle-restricted OXPHOS deficiency associated with intractable infantile epilepsy and abnormal brain MRI findings.

## 2 Material and methods

### 2.1 Patient case report

**Fig. 1:**
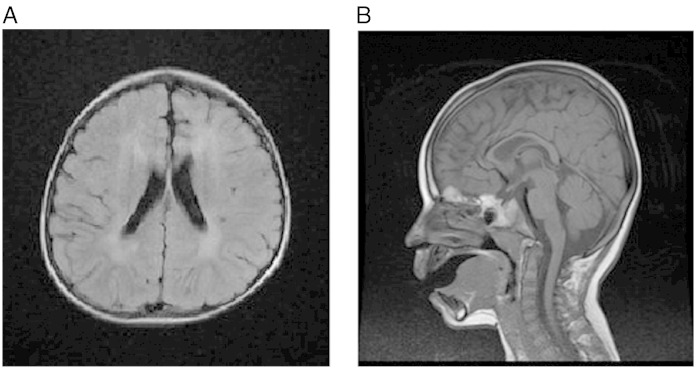
Cranial MRI performed at age 2.5 years. A. Transverse T1 FLAIR image illustrating symmetrical, anterior predominant, white matter signal changes. B. Sagittal T1 weighted image demonstrating thinning of the anterior and mid portions of the corpus callosum.

This young boy is the first child of healthy non-consanguineous, white British parents. He was born at term following an uneventful pregnancy weighing 3132 g (9th–25th centile). Early development was thought to be normal. At approximately 6 months of age he developed tonic upward eye deviation associated with flexion of his arms and neck consistent with infantile spasms. An electroencephalograph (EEG) at this time was grossly abnormal (hypsarrhythmia) and strongly supported a diagnosis of West Syndrome. Cranial MRI was reported as normal. Prednisolone was prescribed and treated the seizures effectively. Steroids were weaned over 6 weeks and he remained seizure free for a further 6 months. By the age of 1 year, it was apparent that his early developmental progress was not being maintained and that he was functioning at the 6–8 month developmental stage. Seizures returned shortly after his first birthday and were prolonged, frequent and on occasion focal, involving his right arm, leg and right side of face. Clonazepam briefly improved seizure frequency, but subsequently his epilepsy has proved refractory to various combinations of anticonvulsant therapy. Prolonged seizures of more than 60 min have been associated with a stepwise regression in his neurodevelopment. Seizure semiology is now predominantly one of epilepsia partialis continua involving the right side of his face, right arm and right leg. The development of focal seizures and the progressive nature of the condition prompted a second cranial MRI at the age of 2 years 6 months. By contrast with the previous scan, this MRI revealed symmetrical subcortical white matter lesions (Fig. 1A) with thinning of the anterior and genu of the corpus callosum (Fig. 1B).

On examination, the patient had small, round, anteriorly rotated ears and a broad nasal root. He demonstrated no visual awareness but startled to loud noise. Tone was increased in all 4 limbs with internal rotation of both legs at the hips. Reflexes were pathologically brisk. Brief myoclonic jerks were evident throughout the examination.

### 2.2 Structural investigation of the nuclear genome using genome-wide array

Early genetic screening included an Affymetrix Genome-wide Human SNP 6.0 array, which was used to detect DNA copy number changes in our patient. Samples were prepared and processed according to the manufacturer's specifications and analysed using locally-established methods [21]. Array CEL intensity files were loaded into Genotyping Console (Affymetrix UK Ltd.) for analysis. An approximately 88 kb deletion was identified within the short arm of chromosome 6, band p25.1 but no similar deletions were identified in the controls. This reported deletion was interrogated against ~ 5000 control samples using Nexus Copy Number v6.1^TM^ (BioDiscovery Inc.). Quantitative polymerase chain reaction (qPCR) using the SYBR-Green method was used to confirm the reported deletion and determine the inheritance pattern [21]. Identification of the genes within the reported deletion locus was achieved using the UCSC genome browser build GRCh36/hg18 at the time.

### 2.3 *LYRM4* and *FARS2* gene sequencing

Total genomic DNA was obtained using standard methods and the coding region plus intron–exon boundaries of the *LYRM4* and *FARS2* genes were amplified using locus specific primers (sequences provided in Supplemental Table S1). Amplicons were sequenced using the BigDye v3.1 kit and capillary electrophoresed on the ABI3130xl fluorescent sequencing platform (Life Technologies, Warrington, UK). Chromatograms were compared to corresponding GenBank reference sequences, *LYRM4* NM_020408.4; *FARS2* NM_006567.3. All sequence variants were cross-referenced against dbSNP (build 135) then investigated using *in silico* methodologies.

### 2.4 *In silico* prediction tools

Amino acid residue conservation and predicted impact of the novel *FARS2* variant was analysed using Ensembl release 66 [22], Polyphen2 [23], SIFT [24] and AlignGVGD [25].

### 2.5 Mitochondrial genome sequencing

Whole mtDNA genome sequencing was performed on DNA derived from muscle as described previously [26].

### 2.6 Tissue culture manipulations

Primary fibroblast and myoblast cultures were established from skin and muscle biopsies and propagated in a humidified incubator with 5% CO_2_ at 37 °C. Fibroblasts were cultured in Eagle's Minimal Essential Medium supplemented with 2 mM l-glutamine, 1 × non-essential amino acids and 10% foetal calf serum (FCS). Myoblasts were grown in Skeletal Muscle Cell Growth Medium (PromoCell GmbH, Heidelberg Germany) with Supplement Mix (Epidermal Growth Factor, basic Fibroblast Growth Factor, and Insulin), 10% foetal calf serum, 50 μg/ml streptomycin and 50 U/ml penicillin and 2 mM l-glutamine. Lentiviral particles containing wild-type *FARS2* were purchased from Genecopoeia (LP-10240-Lv105-0200-S). Cells were infected overnight, following viral infection, transduced cells were selected with 2 μg/ml puromycin.

### 2.7 Respiratory chain enzyme activity

Activities of the individual respiratory chain complexes (complexes I–IV) and citrate synthase were determined spectrophotometrically as previously described [27].

### 2.8 SDS-PAGE and Western blotting

Cells were harvested then lysed in lysate buffer (50 mM Tris 7.5 pH, 130 mM NaCl, 2 mM MgCl_2,_ 1 mM PMSF and 1% NP-40 (v/v)). Proteins (20–50 μg) were separated by 10% SDS-PAGE and electroblotted onto PVDF membrane (Immobilon-P, Millipore Corporation). Membranes were sequentially probed using the following commercially available antibodies: FARS2 (PTG Labs, 16436-1-AP), Complex II SDHA 70 kDa (MitoSciences, MS204), β-actin (Sigma, A1978), COX I (Molecular probes A6403), COX II (Molecular probes, A6404), NDUFB8 (MitoSciences, MS105), NDUFS3 (MitoSciences, MS110) and NDUFA9 (MitoSciences, MS111). Secondary antibodies were HRP conjugated and detection was by chemiluminescence (ECL + Kit, Amersham) according to the manufacturer's instructions and visualised with PhosphorImager/ImageQuant software (Molecular Dynamics, GE Healthcare).

### 2.9 RNA isolation and high-resolution northern blotting

Total RNA was extracted from cultured cells using TRIzoL^TM^ (Invitrogen). Samples (2 μg) were electrophoresed through 15% denaturing polyacrylamide gels. Separated RNA was electroblotted onto a GeneScreen Plus membrane (PerkinElmer, Beaconsfield, UK) and fixed by UV crosslinking prior to hybridization with radiolabelled probes. Probes for mt-tRNA^Phe^, mt-tRNA^Val^ and mt-tRNA^Leu^ were produced by PCR amplification using the following primers (GenBank accession number for human mtDNA: NC_019290.1): human mt-tRNA^Leu(UUR)^ forward (position 3200–3219) 5′-TATACCCACACCCACCCAAG-3′ and reverse (position 3353–3334) 5′-GCGATTAGAATGGGTACAAT-3′; human mt-tRNA^Phe^ forward (position 552–570) 5′-CCAAACCCCAAAGACACCC-3′ and reverse (position 712–694) 5′-GAACGGGGATGCTTGCATG-3′; human mt-tRNA^Val^ forward (position 1579–1598) 5′-CTGGAAAGTGCACTTGGACG-3′ and reverse (position 1734–1714) 5′-GGGTAAATGGTTTGGCTAAGG-3′. Purified PCR products were labelled with [α-^32^P] dCTP (250 μCi, 3000 Ci/mmol; Perkin Elmer) using random hexamers and free nucleotides were removed by gel filtration. Hybridization was carried out overnight at 42 °C in 5 × SSPE, 1% SDS, 10% (w:v) dextran sulphate, 50% (v:v) formamide and 5 × Denhardt's solution. Membranes were stringently washed before detection of signal by PhosphorImager/ImageQuant software.

### 2.10 Metabolic labelling of mitochondrial protein synthesis

Essentially as previously described [28], growing cells in culture were incubated with ^^35^^S methionine/cysteine (2 mCi, 1175 Ci/mmol; Perkin Elmer) for 2 h in methionine- and cysteine-free DMEM (Sigma) to radiolabel the newly synthesised mitochondrial proteins. Radiolabelling was restricted to mtDNA encoded proteins by including 100 μg/ml emetine hydrochloride (Sigma) in the medium to inhibit cytosolic protein synthesis. A cell lysate (30 μg) was then prepared and separated through 15% SDS-PAGE followed by overnight fixation in 3% (v:v) glycerol, 10% (v:v) glacial acetic acid, 30% (v:v) methanol. The gel was then vacuum dried at 60 °C, placed into a PhosphorImager cassette for 24–120 h and analysed by PhosphorImager/ImageQuant software. It was subsequently rehydrated and Coomassie blue stained to confirm equal loading.

### 2.11 Relative quantification of mtDNA

Total DNA was extracted from cultured myoblasts by standard methods. *MT-ND4* was selected as the mtDNA marker and *18S* as a nuclear DNA marker against which to normalise *ND4* value. The primer sequences were: *ND4* forward 5′-CCATTCTCCTCCTATCCCTCAAC-3′ and reverse 5′-CACAATCTGATGTTTTGGTTAAACTATATTT-3′; and *18S* forward 5′-GTAACCCGTTGAACCCCATT-3′ and reverse 5′-CCATCCAATCGGTAGTAGCG-3′. qPCR was performed (LightCycler® Nano, Roche Diagnostics) using the FastStart Essential DNA Green Master (Roche Diagnostics). C_T_ values were calculated automatically by the software and the relative quantification was determined using the ∆∆C_T_ method.

### 2.12 Analysis of mitochondrial morphology and nucleoids

Myoblasts were grown on 35 mm plates as described earlier. Cells were incubated with 3 μl/ml PicoGreen (Quant-IT^TM^) for 1 h in culture media. Tetramethylrhodamine methyl ester (5 nM TMRM^+^, Invitrogen) was added for the final 15 min of incubation and kept in the assay buffer (135 mM NaCl, 5 mM KCl, 0.4 mM KH_2_PO_4_, 1.3 mM CaCl_2_, 1 mM MgSO_4_, 5.5 mM glucose and 20 mM HEPES; pH 7.4 with NaOH) throughout washing and imaging. Imaging was performed at 63 × magnification with an inverted fluorescence microscope (Axiovert 200 M, Carl Zeiss) equipped with FITC and Texas Red filters.

### 2.13 Expression and purification of human FARS2

*FARS2* amplicons were generated from IMAGE clone 5088776/MGC 31883 (BC021112.1) by standard 35 cycle PCR using a proofreading polymerase (KOD Hot Start Novagen) and the following primers, forward 5′-CACACAGGATCCGCAGAGTGTGCCACCCAAAG-3′ and reverse 5′-CACACAGGATCCAGCCTGAGTGAAGTGGTGAC 3′, incorporating *BamH*I restriction sites (underlined). PCR products were digested prior to ligation into *BamH*I linearized pGEX-6P-1, using a Rapid DNA Ligation Kit (Thermo Scientific) according to manufacturer's instructions. The construct was sequenced to confirm orientation and accuracy. It was used to transform competent *Escherichia coli* Tuner cells (Novagen) and subsequently as a template to generate a mutant copy of *FARS2* by site-directed mutagenesis using the Quikchange procedure (Stratagene). Both fusion proteins were expressed following induction with 1 mM IPTG at 37 °C for 4 h and affinity purified using Glutathione Sepharose™ 4B. Wild-type and mutant FARS2 were released by PreScission protease (GE Healthcare) in elution buffer (0.75 ml PBS, 1 mM DTT, 1 mM EDTA and 48 U PreScission protease).

### 2.14 Template production, in vitro transcription and purification of human mitochondrial tRNA^Phe^

A construct was generated containing the T7 RNA polymerase promoter sequence, followed by the hammerhead ribozyme sequence, upstream of the mt-tRNA^Phe^ sequence after which was positioned a *BstN*I site. This was achieved using the following overlapping primers: forward 5′-GTAATACGACTCACTATGGGAGATCTGCTGATGAGTCCGTGAGGACGA AACGGTACCCGGTACCGTCGTTTATGTAGCTTACCTC-3′ and reverse 5′-GTCCTGGTGTTTATGGGGTGATGTGAGCCCGTCTAAACATTTTCAGTGTATTGCTTTGAGGAGGTAAGCTACATAAAC-3′. Following annealing and extension, this double stranded product was cloned into *Sma*I linearized pUC18 and used to transform *E. coli* α-select chemically competent cells (Bioline). Plasmid DNA was then purified and sequenced to validate the construct. This was then linearised with *BstN*I and in vitro transcribed using AmpliScribe™ T7-*Flash*™ Transcription Kit according to the manufacturer's instructions (Epicentre, Madison, WI). RNA was incubated (1 h at 55 °C) in buffer containing 30 mM MgCl_2_ and 40 mM Tris–HCl (pH 8.0) to facilitate self-cleavage by the hammerhead ribozyme. This ensured the exact mt-sequence at the 5′ terminus rather than the T7 consensus motif. Purified RNA was separated by 15% denaturing PAGE. Fully cleaved mt-tRNA^Phe^ was recovered from the gel in elution buffer (1 M NH_4_OAc, 2 mM EDTA and 0.2% (w/v) SDS).

### 2.15 Functional analysis of recombinant mutant FARS2

In vitro aminoacylation assays were performed essentially as described [29], in a 25 μl volume at 30 °C for 20 min using 50 μM ^^3^^H phenylalanine (1 mCi, 126.2 Ci/mmol; Perkin Elmer), 4 μg tRNA and 2–4 μg enzyme in buffer containing 50 mM Tris–HCl pH 7.75, 12 mM MgCl_2_, 10 mM KCl, 0.2 μg/ml BSA and 2.5 mM ATP as described. Prior to addition, tRNAs were heated for 2 min at 60 °C and cooled down slowly to room temperature. At different time points, 5 μl aliquots were spotted onto Whatman 0.45 μM nitrocellulose membrane and precipitated in ice cold 5% trichloroacetic acid. Incorporation of radioactive phenylalanine was measured by liquid scintillation counting. Furthermore, to assess ATP binding only, reactions under similar conditions were performed using 2 mM γATP (250 μCi, 3000 Ci/mmol; Perkin Elmer) and 4 μg enzyme. Incorporation of radioactive ATP was measured by Cerenkov counter.

## 3 Results

### 3.1 Cytogenetic and molecular genetic analysis

**Fig. 2:**
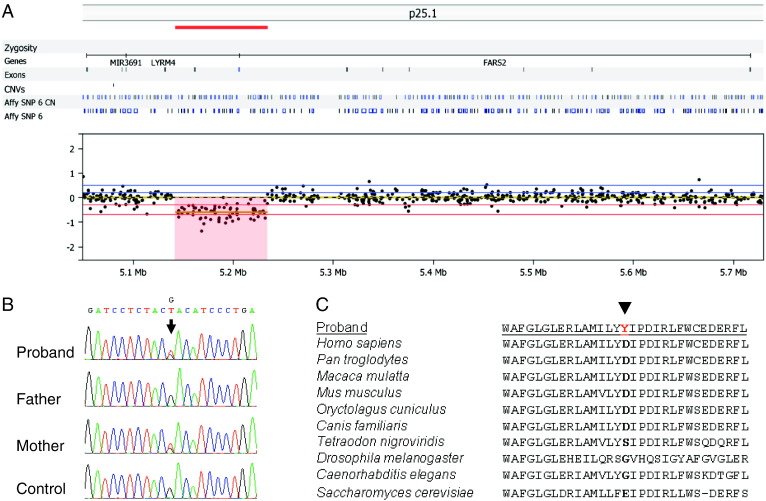
Molecular characterisation of novel *FARS2* mutations. A. Heterozygous 88 kb deletion identified on Array CGH; B. Sequencing chromatograms, arrow indicates the maternally transmitted novel c.973G > T, p.Asp325Tyr *FARS2* variant. C. Alignment of the FARS2 protein sequence flanking the position of the amino acid change from eukaryotic organisms (yeast, worms, flies, fish and mammals) indicating the conservation of the p.Asp325 amino acid.

Karyotyping revealed no abnormalities, whilst high resolution array CGH of the index case revealed an interstitial subtelomeric 6p25.1 deletion, sized at 88 kb, with analysis of familial samples confirming paternal (and grandpaternal) inheritance. Analysis of the breakpoints, chr6:5193613–5281294 (hg19), revealed two RefSeq genes within the locus, *LYRM4* and *FARS2*, both of which are partially deleted — the 3′ exons of *LYRM4* and the regulatory region of the *FARS2* gene, including the promoter and untranslated exon 1 (Fig. 2A). Subsequent molecular genetic analysis of the *LYRM4* gene revealed no known or potentially pathogenic mutations, whilst a novel heterozygous c.973G > T transversion was identified in exon 5 of *FARS2*, predicting a p.Asp325Tyr amino acid substitution. Analysis of parental samples confirmed maternal inheritance of the c.973G > T variant, supporting recessive inheritance in this family (Fig. 2B). Conservation analysis revealed the p.Asp325 residue shows a moderate degree of evolutionary conservation (Fig. 2C), whilst *in silico* predictions using PolyPhen, AlignGVGD and SIFT support a deleterious effect on protein function. Analysis of the mitochondrial genome revealed no known or potentially pathogenic mtDNA mutations, thereby excluding the possibility of a novel or maternally transmitted mtDNA mutation. In particular, no mutation was found within the *MTTF* gene encoding the mt-tRNA^Phe^ molecule.

### 3.2 Biochemical analysis of OXPHOS activities revealed differential expression in patient cell types

**Fig. 3:**
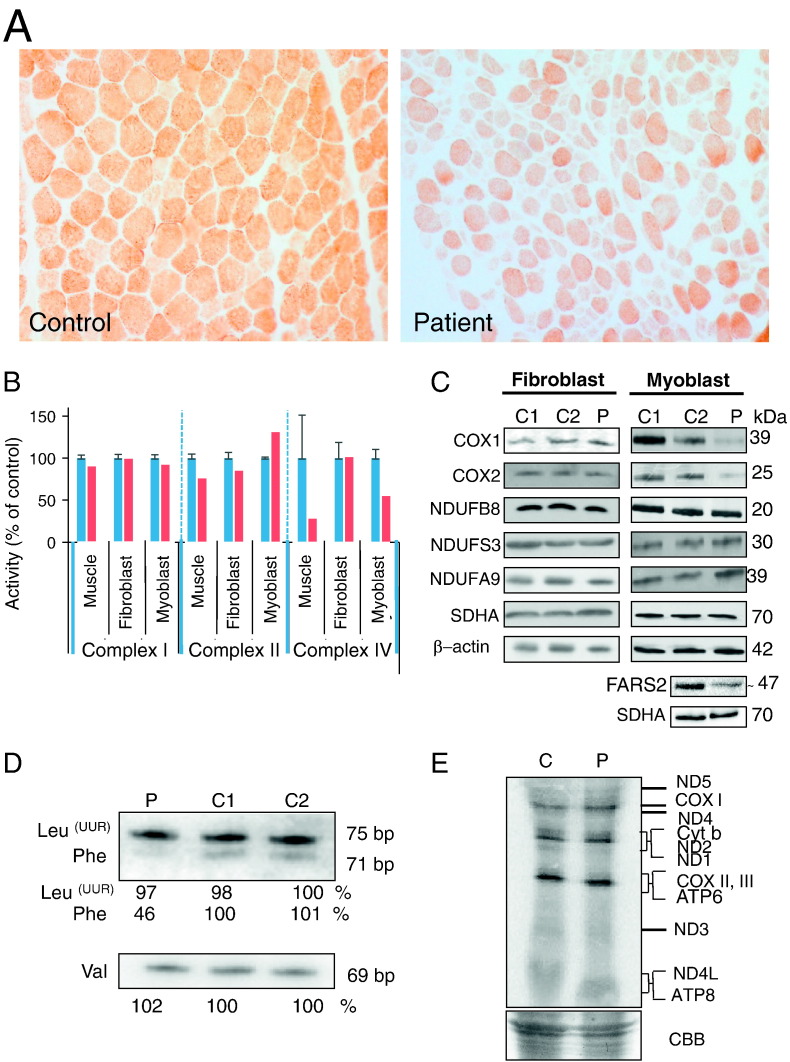
Effect of *FARS2* mutation on mitochondrial homeostasis. A. Cytochrome *c* oxidase (COX) histochemistry of the patient muscle showed a generalised loss of enzyme activity compared to age-matched control tissue. B. Respiratory chain enzyme activity in muscle biopsy, fibroblast and myoblast: activities of complex I, complex II, and complex IV were determined in control (blue) and patient (red) and normalised to citrate synthase. Results are based on three independent measurements and are shown as percent of the mean control value ± standard deviation C. Steady state levels of RC proteins in fibroblasts (left panel) and myoblasts (right panel) were determined by Western blotting. 10% SDS-PAGE was performed with cell lysates (30 μg) from control (C1, C2) and patient (P), except for FARS2 and SDHA in the bottom 2 panels where 80 μg mitochondrial protein was loaded per lane. Western blots were decorated with antibodies to the proteins indicated. Secondary α-antibodies were HRP conjugated and detection was by ECL + and *ImageQuant* software. D. High resolution northern blot analysis was performed on total RNA (2 μg) from control (C1, C2) and patient (P). Membranes were hybridised with radiolabelled probes for mt-tRNA^Phe^, mt-tRNA^Val^ and mt-tRNA^Leu(UUR)^. Densitometric analyses were performed on all blots. A representative example is presented with the values relative to controls for the signals derived for each tRNA below the sample. E. *De novo* mitochondrial protein synthesis in control myoblast (lane C) and the patient myoblast cells (lane P). Designation of proteins is as described by [28]. A section of Coomassie blue (CBB) stained gel is shown indicate equal loading.

A diagnostic skeletal muscle biopsy was obtained from the patient to allow the assessment of respiratory chain enzyme activities. Histochemical analysis revealed decreased reactivity for cytochrome *c* oxidase (COX, or complex IV) (Fig. 3A), an observation confirmed by the spectrophotometric assay of OXPHOS components that showed complex IV to be markedly decreased in the patient's muscle compared to controls (Fig. 3B, Table 1).

Primary fibroblast and myoblast cell lines were established from the patient, permitting a more detailed investigation of the molecular phenotype and characterisation of both the biochemical defect and underlying molecular disease mechanism. Following the spectrophotometric analysis of skeletal muscle, the next assays focussed on the measurement of OXPHOS complex activities in patient-derived cell lines. These appeared to indicate a tissue-specific defect. The respiratory chain enzyme activities were normal in patient fibroblasts, whilst patient myoblasts demonstrated an isolated complex IV defect, recapitulating the observations in skeletal muscle, albeit with a lesser severity (Fig. 3B, Table 1). We next examined the expression of FARS2 protein and several respiratory chain components. Western blot analysis demonstrated that the steady state levels of FARS2 was reduced in the patient. Most mt-aaRSs appear to be present at low levels and so isolated mitochondria rather than cell lysates were analysed for steady state levels of FARS2 protein. The patient exhibited levels that were approximately 50% of control, consistent with expression from only the maternally inherited allele (Fig. 3C). Interestingly the p.Asp325Tyr mutation did not appear to affect stability. The mitochondrially-encoded COXI and COXII subunits of complex IV were normal in patient fibroblasts but decreased in patient myoblasts when compared to age-matched controls (Fig. 3C). In contrast, normal levels of the complex I protein NDUFB8, which is sensitive to CI assembly defects, was observed in both fibroblasts and myoblasts. These findings were consistent with the pattern of respiratory chain enzyme deficiency — an isolated complex IV defect associated with the *FARS2* mutations, only seen in the patient's mature muscle or myoblasts (Fig. 3B, Table 1).

### 3.3 *FARS2* mutations affected mt-tRNA^Phe^ steady state levels in myoblasts.

To study the effect of the *FARS2* mutations on the steady state levels of mt-tRNA^Phe^, high resolution northern blotting was performed using total RNA extracted from patient myoblasts. The levels of mt-tRNA^Val^ and mt-tRNA^Leu(UUR)^ were used as loading controls and the level of mt-tRNA^Phe^ was assessed as a percentage relative to controls. We observed an approximately 54% reduction in the level of mt-tRNA^Phe^ when compared with normal controls (Fig. 3D).

### 3.4 *De novo* mitochondrial protein synthesis was not affected by the *FARS2* mutations

To determine whether the *FARS2* mutations led to impaired mitochondrial protein synthesis, we next evaluated the incorporation of ^^35^^S-methionine/cysteine into *de novo* synthesised mitochondrial proteins in patient myoblasts, compared to age-matched control cells. Although the *FARS2* mutant cells showed a reduction in steady state levels of both complex IV subunits and mt-tRNA^Phe^, no significant decrease was observed in the *de novo* synthesis of mitochondrial proteins (Fig. 3E).

### 3.5 Altered distribution of mitochondrial nucleoids

**Fig. 4:**
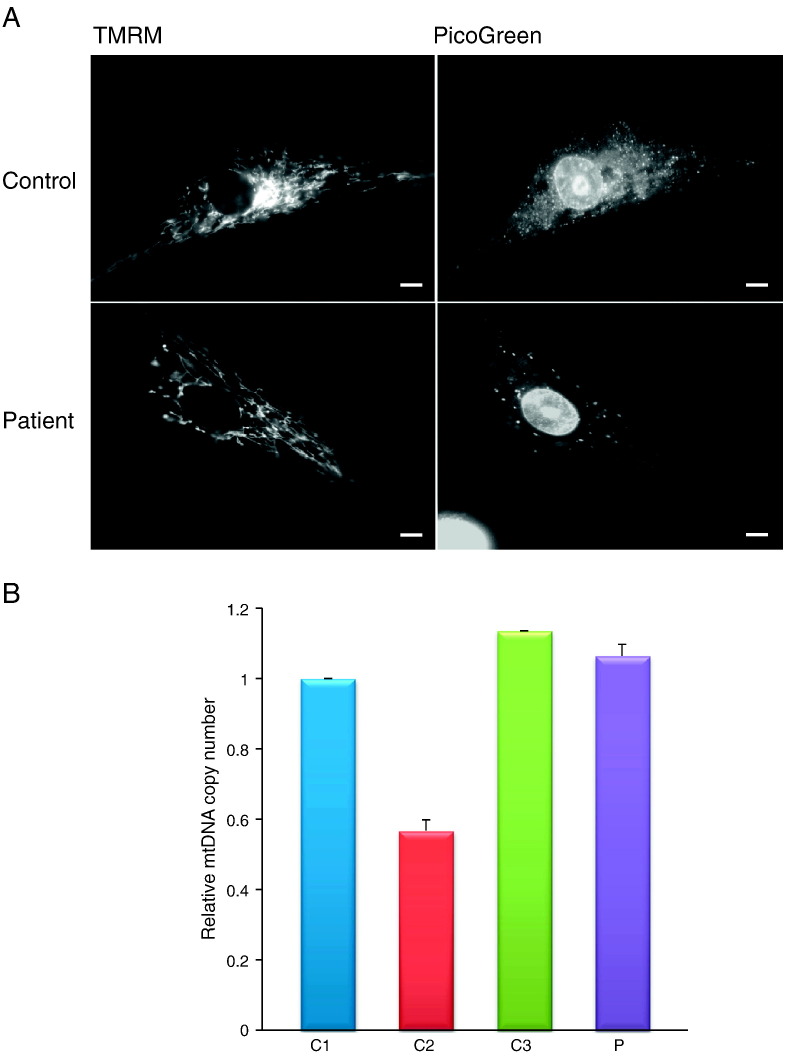
Mitochondrial morphology, mtDNA and nucleoid distribution. A. Nucleoid and mitochondrial morphology imaging was performed with TMRM and PicoGreen in control and patient myoblasts. TMRM accumulated in the mitochondria allowing visualisation of the mitochondrial network while PicoGreen staining localised to nucleoids indicating the position/distribution of non-supercoiled DNA, scale bar = 5 μm B. Relative quantification of mtDNA copy number in controls and patient myoblasts: quantitative real-time PCR was performed using 10 ng of total DNA for three controls (blue, red and green) and the patient (violet). *MT-ND4* and *18S* probes were used for the quantification of the mitochondrial and the nuclear copy number. Results shown are the mean of three measurements from three independent DNA preparations.

The dynamic mitochondrial network and the distribution of nucleoids within were examined in patient myoblasts using fluorescence microscopy following TMRM and PicoGreen staining. No observable differences were found in either mitochondrial morphology or number (Fig. 4A). The distribution of the mitochondrial reticulum appeared normal in patient cells compared to controls. We did observe, however, that nucleoids were consistently found to be both larger and fewer in patient myoblasts (Fig. 4A). After assessing the mtDNA copy number it was clear that this change in nucleoid presentation was not associated with an observable decrease in mtDNA, as the levels in patient cells fell within normal limits (Fig. 4B).

### 3.6 Functional analysis of p.Asp325Tyr recombinant FARS2 indicated a significant decrease in both aminoacylation capacity and ATP binding

**Fig. 5:**
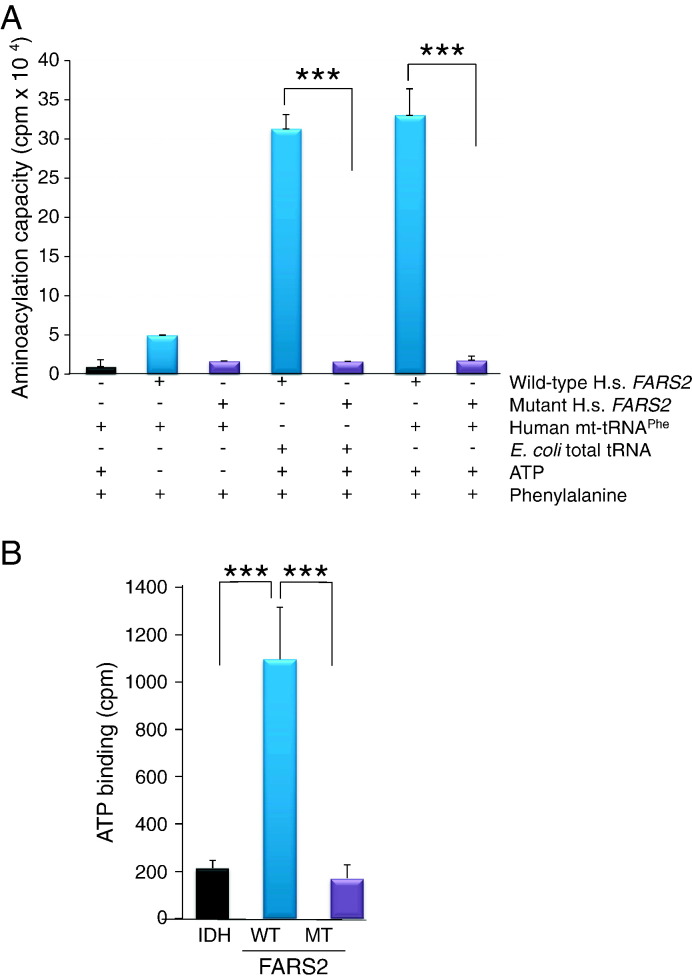
Aminoacylation activity and ATP binding ability of wild-type and mutant FARS2. A. Wild-type (blue) and p.Asp325Tyr mutant (violet) FARS2 proteins were assessed for activity in the presence or absence of ATP using either *E. coli* or human mt-tRNA a substrate. Incorporation of radioactive phenylalanine was measured by liquid scintillation counting n = 4. B. Wild-type (blue) and mutant (violet) FARS2 protein were assessed for ability to bind ATP. Isocitric dehydrogenase (IDH, black) was used as a control. Incorporation of radioactive ATP was measured by Cerenkov counter, n = 3.

To investigate if the *FARS2* mutation in the patient cells was responsible for compromised mt-tRNA aminoacylation efficiency, we assessed in vitro aminoacylation activity of a recombinant FARS2 protein engineered to carry the identical p.Asp325Tyr missense mutation as observed in our patient. Both the mutant and wild-type recombinant proteins were assessed for monodispersion prior to activity assays. Wild type FARS2 showed good activity on both the bacterial tRNA and human mt-tRNA^Phe^. As expected this activity was ATP dependent (Fig. 5A). In contrast, mutant FARS2 protein showed no detectable activity on either bacterial or human mt-tRNA^Phe^ substrate even in the presence of ATP (Fig. 5A). In addition, we examined the previously reported crystal structure of FARS2 [30], upon which we superimposed the predicted structure of the p.Asp325Tyr mutant. The modest distortion suggested that the *FARS2* mutation would widen the ATP binding pocket and thus decrease affinity for ATP (Fig. S1). Therefore, we further assessed the ability of both mutant and wild-type FARS2 to bind ATP in vitro. As predicted, wild type FARS2 protein showed high levels of ATP binding in contrast to the mutant p.Asp325Tyr FARS2 protein, which showed no detectable ATP binding (Fig. 5B).

### 3.7 Transduction of patient myoblasts restored the steady state level of both OXPHOS polypeptides and mt-tRNA^Phe^

**Fig. 6:**
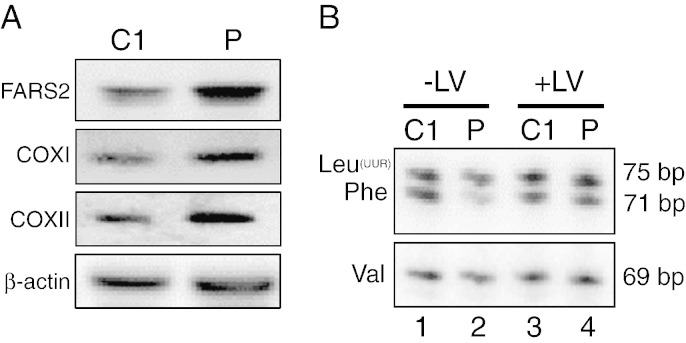
Lentiviral transduction of myoblasts with wildtype *FARS2*. Both control (C1) and patient (P) myoblasts were transduced with lentivirus designed to express wildtype *FARS2*. Images are representative of a minimum of n = 3. A. Following selection cell lysates (40 μg) were subjected to Western blot analysis to determine the levels of FARS2 and mt-encoded complex IV proteins. B. High resolution northerns were performed on RNA from control (C1) and patient (P) prior to (− LV; lanes 1 and 2) or post (+ LV; lanes 3 and 4) lentiviral transduction with wild type *FARS2*.

Immortalised patient and control myoblasts were transduced with a lentiviral construct that would express wild type FARS2. Following antibiotic selection to propagate transduced cells, RNA and cell lysates were prepared. Similar analyses to those already described were performed on cell lines following transduction to examine steady state levels of both OXPHOS polypeptides and mt-tRNAs. Although Western blot analysis is at best semi-quantitative, steady state levels of mitochondrial aminoacyl tRNA synthetases does generally appear to be very low requiring high amounts of mitochondrial protein to allow detection [31]. The transduction procedure was performed simultaneously for both control and patient myoblasts, and although the level of FARS2 expression became significantly increased in both lines, the expression levels appeared to be even higher in the patient (Fig. 6A, *cf* C1 *vs* P). Analysis of steady state levels of mitochondrially encoded COXI and COXII, which were lower than control in untransduced cells, were now restored, even exceeding control levels. Similarly following transduction, analysis of mt-tRNA^Phe^ levels indicated an increase from ~ 46% to > 90% relative to control (Fig. 6B *cf* lanes 2 and 4). Transduction appeared to leave levels of mt-tRNA^Val^ and mt-tRNA^Leu(UUR)^ unchanged (Fig. 6B). Together these data indicate that replacement of wild type FARS2 was sufficient to restore levels of mt-tRNA^Phe^ and complex IV polypeptides.

## 4 Discussion

We have identified a patient with early-onset epilepsy and isolated complex IV deficiency due to mutations in the *FARS2* gene, prompted by diagnostic array CGH studies that identified a heterozygous interstitial 6p25.1 deletion. Scrutiny of the genes within the 88 kb deleted region revealed two genes, *LYRM4* and *FARS2*, 1both predicted to have a mitochondrial function. *LYRM4* encodes a protein required for nuclear and mitochondrial iron–sulphur protein biosynthesis, whilst *FARS2* encodes the mitochondrial phenylalanyl-tRNA synthetase. At the time of referral, neither the *LYRM4* or *FARS2* genes had been reported in association with human pathology therefore both genes were bi-directionally sequenced. Since then a mutation in *LYRM4* has been identified in patients with combined OXPHOS disease [32]. The affected individuals were homozygous for the mutation and manifested with deficiencies in respiratory chain complexes I, II and III, as well as aconitase and ferrochelatase, all of which contain iron sulphur clusters [32]. This presentation differed to our patient and no potentially pathogenic mutations were identified within the *LYRM4* gene. Analysis of the *FARS2* gene, however, revealed a novel heterozygous c.973G > T transversion, predicting a p.Asp325Tyr amino acid substitution. Whole mtDNA genome sequencing was undertaken and excluded the possibility of a co-existing mtDNA point mutation, particularly within the mt-tRNA^Phe^ (*MTTF*) gene. Investigations into the consequences of a *FARS2* mutation were therefore pursued.

The *FARS2* protein has four major domains: an N-terminal region (residues 1–83), a catalytic domain (residues 84–325), a linker region (residues 326–358) and a C-terminal domain (residues 359–451). The maternally-transmitted p.Asp325Tyr *FARS2* mutation is located in the catalytic domain where the aminoacylation reaction occurs. Based on the reported crystal structure of FARS2 [30], we predict that this mutation will disrupt ATP binding, affecting aminoacylation efficiency. Consistent with an inability to bind ATP, we found a significant reduction in the aminoacylation capacity of the recombinant mutant protein in vitro, where activity was essentially undetectable. This was also consistent with the complex IV deficiency observed in the patient's muscle biopsy, the decreased steady state levels of COXI and COXII protein expression in myoblasts and in the reduced steady-state levels of the mt-tRNA^Phe^ transcript. These low levels of protein and mt-tRNA were relieved by transduction with a wild type copy of FARS2 confirming that the mutation was responsible for the biochemical phenotype and clinical presentation.

Unlike many mitochondrial disorders, where there is often vast clinical heterogeneity with a given genotype, a rapidly increasing number of mitochondrial tRNA synthetase genes are being reported in association with very discrete clinical phenotypes and syndromes. For example, *DARS2* mutations are associated with LBSL [7], *YARS2* mutations with MLASA [10] and *RARS2* mutations with PCH6 [9]. Interestingly, a recent report describes novel *FARS2* mutations in two families with fatal intractable myoclonic epilepsy [20]; whilst the initial presenting symptom clearly correlates with our index case, there was no evidence of marked cerebral atrophy in our patient's brain MRI, which was a striking feature in the cases presented by these authors, although our patient did have structural brain abnormalities involving the corpus callosum and white matter abnormalities.

Clinical phenotypes caused by mutations in the specific mt-aminoacyl-tRNA synthetases are associated with diversity in both tissue-specificity and clinical presentation [6]. Variability in clinical presentation may suggest the involvement of these enzymes in a secondary function besides their primary role in mitochondrial protein synthesis, however, these have not yet been identified. Tissue-specificity may arise due to variation in the level of gene expression between tissues, which will designate that the enzyme activity will be either above or below the critical threshold in a particular tissue. In addition, differences in requirements for respiratory chain complexes activity and ATP produced by oxidative phosphorylation may also lead to the presence or absence of the phenotype in a particular tissue. In our patient, the respiratory chain complex IV deficiency was observed in both mature muscle and myoblasts but not in patient fibroblasts; this is not unprecedented and has been observed in other mt-aminoacyl-tRNA synthetase defects including *YARS2* [11]. Moreover, we might have expected to observe multiple respiratory chain defects in these tissues, rather than an isolated complex IV defect, given the role of mt-aminoacyl-tRNA synthetases in mitochondrial translation; again, variability in the biochemical defects observed in human mt-aminoacyl-tRNA synthetase disorders has been reported, with *MARS2* mutations leading to isolated complex I deficiency [16]. There is no clear explanation for this and it does not appear to be related to the phenylalanine content of mtDNA-encoded COX subunits (Supplemental Table S2). Despite the biochemical defects observed in myoblasts, we were not able to demonstrate a marked deficiency in *de novo* mitochondrial protein synthesis. This may, however, reflect a low level of residual aminoacylation activity that is sufficient to promote elongation under experimental conditions, where cells are essentially glycolytic.

To the best of our knowledge, the identification of a microdeletion represents a novel disease mechanism in association with a primary mitochondrial presentation; 6p25 deletions represent a rare but clinically characterised cytogenetic microdeletion syndrome, with less than 15 interstitial deletion cases reported in the literature to date [33,34]. The smallest deletion reported in association with the 6p25 deletion syndrome is 900 kb and involves the *CDYL*, *RPP40*, *PP1R3G*, *LYRM4* and *FARS2* genes [33]. Bozza et al. have hypothesised that these genes are responsible for the characteristic 6p25 deletion syndrome features, given that the phenotype correlates to that of other deletion syndrome cases, whose deletions span up to 500 Mb of the 6p25 region. Our patient presented with intractable infantile epilepsy (a feature never before reported in association with the 6p25 microdeletion syndrome) and a much smaller deletion than previously reported thereby prompting further investigation of the affected genes for a possible recessive aetiology. Our patient is reported to have minor dysmorphic features involving the nasal root and the ears, whilst these dysmorphic features have not previously been reported in association with recessively-inherited *FARS2* defects, they have been reported in patients with a classic 6p25 microdeletion syndrome. These findings suggest that haploinsufficiency of the *LYRM4* and *FARS2* genes may be, at least in part, responsible for some of the dysmorphic features of the 6p25 deletion syndrome.

In conclusion, we demonstrate that compound heterozygous *FARS2* mutations lead to an early-onset mitochondrial disease phenotype associated with respiratory chain dysfunction and characterised by infantile spasms that evolved into refractory focal epilepsy, neurodevelopmental regression and the development of subcortical white matter abnormalities on cranial MRI. Given the increasing influence of targeted exome capture and next generation sequencing to identify novel genes associated with early-onset mitochondrial disease, including several mt-aminoacyl-tRNA synthetases such as *AARS2* [14] and *EARS2* [15], our study nicely demonstrates that routine diagnostic genetic testing methodologies including array CGH can be crucial in the identification of underlying genetic defects. This may be of particular relevance to patients who have an as yet undetermined genetic defect following exome sequencing and principally those in whom single heterozygous nuclear gene defects have been identified.

The following are the supplementary data related to this article.

Fig. S1 The structure of wild type and mutated p.Asp325Tyr FARS2 protein.
A. The structure of the wildtype FARS2 (PDB reference 3CMQ) is depicted in grey. The ATP binding residues are shown in red and boxed; Asp325 position is indicated in yellow. B. ESYPred3D software was used to predict the change in structure once the p.Asp325Tyr mutation was introduced into FARS2, shown in cyan with ATP binding residues in red and boxed; Tyr325 is shown in yellow. C. The two structures were superimposed using a number of methods all of which gave the same modest changes shown here using the Chimera 1.7 software.

Table S1 Oligonucleotide sequences designed for *LYRM4* (NM_020408.4) and *FARS2* (NM_006567.3) gene analysis.

Table S2 Phenylalanine content of mtDNA encoded polypeptides.
The % phenyalanine content does not directly correlate with the deficiency seen in respiratory chain complex activities.

## Figures and Tables

**Table 1:** Spectrophotometric activity measurements of OXPHOS complexes.

	Skeletal muscle biopsy		Fibroblast		Myoblast	
	Patient	Controls	Patient	Controls	Patient	Controls
		(Mean ± SD, n = 25)		(Mean ± SD, n = 8)		(Mean ± SD, n = 4)
Complex I	0.093	0.104	0.195	0.197	0.139	0.151
nmol NADH oxidised·min^− 1^·unit citrate synthase^− 1^		± 0.036		± 0.043		± 0.042
Complex II	0.110	0.145	0.187	0.219	0.165	0.126
nmol DCPIP reduced·min^− 1^·unit citrate synthase^− 1^		± 0.047		± 0.067		± 0.013
Complex IV	0.318	1.124	1.081	1.083	0.601	1.084
× 10^− 3^ K s^− 1^·unit citrate synthase^− 1^		± 0.511		± 0.186		± 0.105

## References

[1] Taylor R.W., Turnbull D.M. (2005). Mitochondrial DNA mutations in human disease. Nat. Rev. Genet..

[2] Tuppen H.A., Blakely E.L., Turnbull D.M., Taylor R.W. (2010). Mitochondrial DNA mutations and human disease. Biochim. Biophys. Acta.

[3] Rötig A. (2011). Human diseases with impaired mitochondrial protein synthesis. Biochim. Biophys. Acta Bioenerg..

[4] Bonnefond L., Fender A., Rudinger-Thirion J., Giege R., Florentz C., Sissler M. (2005). Toward the full set of human mitochondrial aminoacyl-tRNA synthetases: characterization of AspRS and TyrRS. Biochemistry.

[5] Yadavalli S.S., Klipcan L., Zozulya A., Banerjee R., Svergun D., Safro M., Ibba M. (2009). Large-scale movement of functional domains facilitates aminoacylation by human mitochondrial phenylalanyl-tRNA synthetase. FEBS Lett..

[6] Konovalova S., Tyynismaa H. (2013). Mitochondrial aminoacyl-tRNA synthetases in human disease. Mol. Genet. Metab..

[7] Scheper G.C. (2007). Mitochondrial aspartyl-tRNA synthetase deficiency causes leukoencephalopathy with brain stem and spinal cord involvement and lactate elevation. Nat. Genet..

[8] Synofzik M., Schicks J., Lindig T., Biskup S., Schmidt T., Hansel J., Lehmann-Horn F., Schöls L. (2011). Acetazolamide-responsive exercise-induced episodic ataxia associated with a novel homozygous DARS2 mutation. J. Med. Genet..

[9] Edvardson S., Shaag A., Kolesnikova O., Gomori J.M., Tarassov I., Einbinder T., Saada A., Elpeleg O. (2007). Deleterious mutation in the mitochondrial arginyl-transfer RNA synthetase gene is associated with pontocerebellar hypoplasia. Am. J. Hum. Genet..

[10] Riley L.G. (2010). Mutation of the mitochondrial tyrosyl-tRNA synthetase gene, <i>YARS2</i> causes myopathy, lactic acidosis, and sideroblastic anemia—MLASA Syndrome. Am. J. Hum. Genet..

[11] Sasarman F., Nishimura T., Thiffault I., Shoubridge E.A. (2012). A novel mutation in YARS2 causes myopathy with lactic acidosis and sideroblastic anemia. Hum. Mutat..

[12] Belostotsky R. (2011). Mutations in the mitochondrial seryl-tRNA synthetase cause hyperuricemia, pulmonary hypertension, renal failure in infancy and alkalosis, HUPRA syndrome. Am. J. Hum. Genet..

[13] Pierce S.B., Chisholm K.M., Lynch E.D., Lee M.K., Walsh T., Opitz J.M., Li W., Klevit R.E., King M.-C. (2011). Mutations in mitochondrial histidyl tRNA synthetase HARS2 cause ovarian dysgenesis and sensorineural hearing loss of Perrault syndrome. Proc. Natl. Acad. Sci..

[14] Gotz A. (2011). Exome sequencing identifies mitochondrial alanyl-tRNA synthetase mutations in infantile mitochondrial cardiomyopathy. Am. J. Hum. Genet..

[15] Steenweg M.E. (2012). Leukoencephalopathy with thalamus and brainstem involvement and high lactate ‘LTBL’caused by EARS2 mutations. Brain.

[16] Bayat V. (2012). Mutations in the mitochondrial methionyl-tRNA synthetase cause a neurodegenerative phenotype in flies and a recessive ataxia (ARSAL) in humans. PLoS Biol..

[17] Pierce S.B., Gersak K., Michaelson-Cohen R., Walsh T., Lee M.K., Malach D., Klevit R.E., King M.C., Levy-Lahad E. (2013). Mutations in LARS2, encoding mitochondrial leucyl-tRNA synthetase, lead to premature ovarian failure and hearing loss in Perrault syndrome. Am. J. Hum. Genet..

[18] Santos-Cortez R.L. (2013). Mutations in KARS, encoding lysyl-tRNA synthetase, cause autosomal-recessive nonsyndromic hearing impairment DFNB89. Am. J. Hum. Genet..

[19] Shamseldin H.E., Alshammari M., Al-Sheddi T., Salih M.A., Alkhalidi H., Kentab A., Repetto G.M., Hashem M., Alkuraya F.S. (2012). Genomic analysis of mitochondrial diseases in a consanguineous population reveals novel candidate disease genes. J. Med. Genet..

[20] Elo J.M. (2012). Mitochondrial phenylalanyl-tRNA synthetase mutations underlie fatal infantile Alpers encephalopathy. Hum. Mol. Genet..

[21] Tyreman M., Abbott K.M., Willatt L.R., Nash R., Lees C., Whittaker J., Simonic I. (2009). High resolution array analysis: diagnosing pregnancies with abnormal ultrasound findings. J. Med. Genet..

[22] Flicek P. (2012). Ensembl 2012. Nucleic Acids Res..

[23] Adzhubei I.A., Schmidt S., Peshkin L., Ramensky V.E., Gerasimova A., Bork P., Kondrashov A.S., Sunyaev S.R. (2010). A method and server for predicting damaging missense mutations. Nat. Methods.

[24] Kumar P., Henikoff S., Ng P.C. (2009). Predicting the effects of coding non-synonymous variants on protein function using the SIFT algorithm. Nat. Protoc..

[25] Mathe E., Olivier M., Kato S., Ishioka C., Hainaut P., Tavtigian S.V. (2006). Computational approaches for predicting the biological effect of p53 missense mutations: a comparison of three sequence analysis based methods. Nucleic Acids Res..

[26] Taylor R.W. (2003). Mitochondrial DNA mutations in human colonic crypt stem cells. J. Clin. Investig..

[27] Kirby D.M., Thorburn D.R., Turnbull D.M., Taylor R.W., Liza A.P., Eric A.S. (2007). Biochemical assays of respiratory chain complex activity.

[28] Chomyn A. (1996). In vivo labeling and analysis of human mitochondrial translation products. Methods Enzymol..

[29] Sohm B., Frugier M., Brule H., Olszak K., Przykorska A., Florentz C. (2003). Towards understanding human mitochondrial leucine aminoacylation identity. J. Mol. Biol..

[30] Klipcan L., Levin I., Kessler N., Moor N., Finarov I., Safro M. (2008). The tRNA-induced conformational activation of human mitochondrial phenylalanyl-tRNA synthetase. Structure.

[31] Rorbach J., Yusoff A.A., Tuppen H., Abg-Kamaludin D.P., Chrzanowska-Lightowlers Z.M., Taylor R.W., Turnbull D.M., McFarland R., Lightowlers R.N. (2008). Overexpression of human mitochondrial valyl tRNA synthetase can partially restore levels of cognate mt-tRNAVal carrying the pathogenic C25U mutation. Nucleic Acids Res..

[32] Lim S.C. (2013). Mutations in LYRM4, encoding iron-sulfur cluster biogenesis factor ISD11, cause deficiency of multiple respiratory chain complexes. Hum. Mol. Genet..

[33] Bozza M., Bernardini L., Novelli A., Brovedani P., Moretti E., Canapicchi R., Doccini V., Filippi T., Battaglia A. (2013). 6p25 Interstitial deletion in two dizygotic twins with gyral pattern anomaly and speech and language disorder. Eur. J. Paediatr. Neurol..

[34] DeScipio C. (2007). The 6p subtelomere deletion syndrome. Am. J. Med. Genet. C: Semin. Med. Genet..

