# Supplementary figures and images for: Mutation of the human mitochondrial phenylalanine-tRNA synthetase causes infantile-onset epilepsy and cytochrome c oxidase deficiency
Source: Biochim Biophys Acta. 2014 Jan;1842(1):56–64. doi: 10.1016/j.bbadis.2013.10.008 (PMC3898479; doi:10.1016/j.bbadis.2013.10.008)

## Slide 1
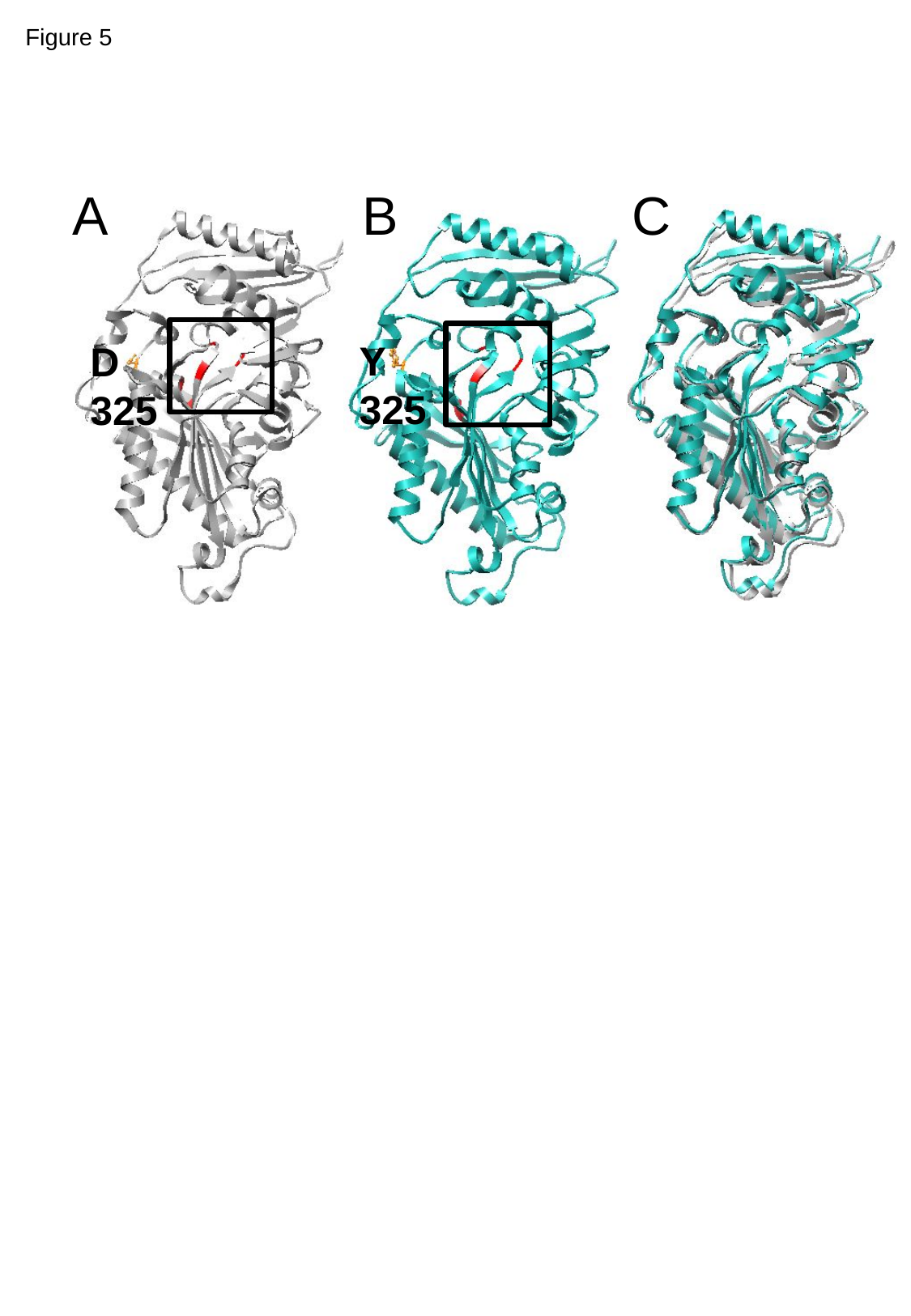

Figure 5
A
D 325
B
Y 325
C

Supplement: Fig. S1 — The structure of wild type and mutated p.Asp325Tyr FARS2 protein. A. The structure of the wildtype FARS2 (PDB reference 3CMQ) is depicted in grey. The ATP binding residues are shown in red and boxed; Asp325 position is indicated in yellow. B. ESYPred3D software was used to predict the change in structure once the p.Asp325Tyr mutation was introduced into FARS2, shown in cyan with ATP binding residues in red and boxed; Tyr325 is shown in yellow. C. The two structures were superimposed using a number of methods all of which gave the same modest changes shown here using the Chimera 1.7 software. [file mmc1.pptx]
