# Supplementary material for: Mutation of the human mitochondrial phenylalanine-tRNA synthetase causes infantile-onset epilepsy and cytochrome c oxidase deficiency
Source: Biochim Biophys Acta. 2014 Jan;1842(1):56–64. doi: 10.1016/j.bbadis.2013.10.008 (PMC3898479; doi:10.1016/j.bbadis.2013.10.008)
Supplement: Table S1 — Oligonucleotide sequences designed for LYRM4 (NM_020408.4) and FARS2 (NM_006567.3) gene analysis. [file mmc2.docx]

**Table S1**

Oligonucleotide sequences designed for *LYRM4* (NM_020408.4) and *FARS2* (NM_006567.3) gene analysis

| ***LYRM4***  Exon | Forward | Reverse | Amplicon size (bp) |
| --- | --- | --- | --- |
| 1 | 5’-GAGGCATCTCCCTCCCAGC-3’ | 5’-CAGGAGCACGCTTTTCGATG-3’ | 500 |
| 2 | 5’-TTTGAAAGCATAGCCTCAGACA-3’ | 5’-GCACGGCTGTCTAATCAAGG-3’ | 262 |
| 3a | 5’-ATGTTCCTTCTGGCCTTTCC-3’ | 5’-GGTCAAAGGCTCAGGGAGAT-3’ | 501 |
| 3b | 5’-GCACCCCATTCCTACCTTGT-3’ | 5’-CTTCTGTCCACCAGCCAGAT-3’ | 546 |
| 3c | 5’-AGAGAGCCCCAGCCTGAC-3’ | 5’-CCCCAGATGACAAGCAAGTT-3’ | 370 |

| ***FARS2***  Exon | Forward | Reverse | Amplicon size (bp) |
| --- | --- | --- | --- |
| 1a | 5’-TGGGAGATGCAAAGAACACA-3’ | 5’-CGTGACCACTGGAGAAAGGT-3’ | 459 |
| 1b | 5’-CAGCAGCATCACCCTCTGT-3’ | 5’-GGCAAAATAAGGCAAGCAAG-3’ | 487 |
| 2 | 5’-TTTCATTACGTTTATTGACAGGACA-3’ | 5’-CCAAAACACTTCTGTCCCAAG-3’ | 372 |
| 3 | 5’-TGATCACAAGAAAGGGCAGA-3’ | 5’-AAGGGAGGGTTCCTTTAGAGC-3’ | 249 |
| 4 | 5’-TGTCAGGGAGTGGTATGAACC-3’ | 5’-CGATCCTTGACAGCCATTTT-3’ | 289 |
| 5 | 5’-AATTAGTCAAGTGCAACTTTTTATGA-3’ | 5’-CACATGACATTTATCACACAGAGA-3’ | 300 |
| 6 | 5’-CTTGTTCATCCCGCACTCAC-3’ | 5’-AAGGAATAGGAAAAGACCTTCCTC-3’ | 394 |
