# Supplementary material for: Mutation of the human mitochondrial phenylalanine-tRNA synthetase causes infantile-onset epilepsy and cytochrome c oxidase deficiency
Source: Biochim Biophys Acta. 2014 Jan;1842(1):56–64. doi: 10.1016/j.bbadis.2013.10.008 (PMC3898479; doi:10.1016/j.bbadis.2013.10.008)
Supplement: Table S2 — Phenylalanine content of mtDNA encoded polypeptides. The % phenyalanine content does not directly correlate with the deficiency seen in respiratory chain complex activities. [file mmc3.docx]

| **mtDNA encoded Polypeptide** | **No. of Phe amino acids** | **% Phe of total residues** |
| --- | --- | --- |
| mtATP6 | 9 | 4 % |
| mtATP8 | 1 | 1.4 % |
| mtND1 | 16 | 5 % |
| mtND2 | 5 | 4.3 % |
| mtND3 | 8 | 7 % |
| mtND4L | 20 | 4.4 % |
| mtND5 | 38 | 6.3 % |
| mtND6 | 10 | 5.7 % |
| mtCOX1 | 41 | 8 % |
| mtCOX2 | 10 | 4.4 % |
| mtCOX3 | 23 | 8.8 % |
| CytB | 24 | 6.3 % |

Table S2 Phenylalanine content of mtDNA encoded polypeptides

The % phenyalanine content does not directly correlate with the deficiency seen in respiratory chain complex activities.
